# An eHealth Intervention for Patients in Rural Areas: Preliminary Findings From a Pilot Feasibility Study

**DOI:** 10.2196/resprot.2861

**Published:** 2014-06-12

**Authors:** Geoffrey Schrader, Niranjan Bidargaddi, Melanie Harris, Lareen Newman, Sarah Lynn, Leigh Peterson, Malcolm Battersby

**Affiliations:** ^1^Mental Health Observatory Research UnitCountry Health SA Local Health NetworkAdelaideAustralia; ^2^e-Health ResearchSchool of MedicineFlinders UniversityBedford ParkAustralia; ^3^Flinders Human Behaviour Health Research UnitSchool of MedicineFlinders UniversityAdelaideAustralia; ^4^Southgate Institute for Health Society & EquitySchool of MedicineFlinders UniversityAdelaideAustralia; ^5^Uniting Care Wesley Port Adelaide IncMt GambierAustralia; ^6^Mount Gambier District Health ServiceCountry Health SA Local Health NetworkMt GambierAustralia

**Keywords:** eHealth, chronic disease, rural health

## Abstract

**Background:**

eHealth facilitation of chronic disease management has potential to increase engagement and effectiveness and extend access to care in rural areas.

**Objective:**

The objective of this study was to demonstrate the feasibility and acceptability of an eHealth system for the management of chronic conditions in a rural setting.

**Methods:**

We developed an online management program which incorporated content from the Flinders Chronic Condition Management Program (Flinders Program) and used an existing software platform (goACT), which is accessible by patients and health care workers using either Web-enabled mobile phone or Internet, enabling communication between patients and clinicians. We analyzed the impact of this eHealth system using qualitative and simple quantitative methods.

**Results:**

The eHealth system was piloted with 8 recently hospitalized patients from rural areas, average age 63 (SD 9) years, each with an average of 5 chronic conditions and high level of psychological distress with an average K10 score of 32.20 (SD 5.81). Study participants interacted with the eHealth system. The average number of logins to the eHealth system by the study participants was 26.4 (SD 23.5) over 29 weeks. The login activity was higher early in the week.

**Conclusions:**

The pilot demonstrated the feasibility of implementing and delivering a chronic disease management program using a Web-based patient-clinician application. A qualitative analysis revealed burden of illness and low levels of information technology literacy as barriers to patient engagement.

## Introduction

eHealth facilitation of chronic disease management has potential to add to program components, increase engagement and effectiveness, and extend access for underserved groups [1-5]. Systems have been developed for specific chronic conditions, particularly diabetes [6], but generic chronic disease management systems are also needed to structure overall care, especially for the majority of patients who have multi-morbidities [7,8]. However, there appear to be no well-evaluated eHealth systems to support delivery of generic chronic disease management and self-management support for individual patients [9]. Comorbidity is the norm in chronic illness, and mental health problems are often present [8,10]. We therefore piloted an eHealth disease management program in people with comorbid mental health and physical health conditions or risk factors who live in rural areas. eHealth technologies should be developed and evaluated from the start as complex individual, social, organizational, and technical interventions [11]. We therefore report quantitative and qualitative data from this pilot, providing preliminary assessment of initial patient acceptance, patient engagement, feasibility of delivery, and outcome measurement to inform further system development.

Our objective was to inform development of an eHealth system of chronic disease management by observing its use by patients with comorbid chronic diseases who live in rural areas and health care workers delivering the program to the patients.

## Methods

### The Intervention

An online management program was specifically developed for the study. This incorporated content from the Flinders Chronic Condition Management Program (Flinders Program) and used an existing software platform (goACT), which is accessible by patients and health care workers using either Web-enabled mobile phone or Internet [12].

The Flinders Program is an overarching program for chronic condition management applicable to medical and psychiatric conditions and to multi-morbidities [7]. It provides a structured collaborative disease management process addressing behaviors of both patient and clinician. The program is based on cognitive behavior therapy, motivational interviewing, and behavioral psychotherapy. It uses a set of tools: the Partners in Health scale (PIH), Cue and Response interview (C&R), and Problem and Goals assessment (P&G). The patient completes the PIH to assess self-management knowledge, attitudes, behaviors, and impacts of chronic conditions. The health care worker uses the C&R to further explore the same concepts and shares his or her perspective with the patient. Strengths, barriers, priorities, and goals identified through shared use of these tools are incorporated into a negotiated care plan that integrates self-management and medical issues, management aims, agreed interventions, responsibilities, and review dates. The Flinders Program care plan tailors a range of possible self-management interventions to the individual, including disease-specific programs, skill-building programs, or community activities. The Flinders care plan is provided to the patient and, with permission, their health professionals and can be incorporated into an overall medical care plan.

Research studies have shown improved outcomes with use of the Flinders Program across a variety of conditions and patient groups [13-16], including patients with mental health disorders [17].

goACT is an online platform accessible by patients and health workers using either Web-enabled mobile phone or computer. Tools for some psychological therapies were already available on the platform but additional forms and communications could readily be added. Investigators wished to assess the feasibility of eHealth delivery of as many components of the Flinders Program as possible. All Flinders Program components (PIH, C&R, and P&G and the Flinders care plan) were therefore added to the goACT platform. Features of electronic systems were used where possible to improve on paper-based methods, for example in transfer of information between components of the program, and continual updating and sharing of information. eHealth features, such as reminders about goals, appointments, and activities, were integrated and negotiated so patients could view them as short message service (SMS) text or email messages or by logging into their goACT webpage. The patient was able to engage with goACT software to record progress and notes against their goals and activities on the Flinders Program and to send messages to the coach. Clients could also access additional goACT tools such as mood diaries.

eHealth-supported delivery of the Flinders Program included completion of PIH, C&R, and P&G tools and care plan into the goACT system where results could be accessed and updated by patients and health care workers, automated delivery of patient supports (such as action and appointment reminders), and email and SMS communication options to supplement any meetings or telephone contact agreed upon between patient and health care worker. The Flinders care plan was shared with health care providers and support network as identified by the patient, using appropriate electronic or physical formats.

### Setting

The study was conducted in 2011 and 2012 and based in Mount Gambier, a regional center in South Australia with a population of about 25,000. The intervention was delivered by staff of a local community care organization, UnitingCare Wesley.

### Participants

Inclusion criteria for the study were the presence of chronic physical and psychological comorbidities as recorded in case records of the recruiting organization, being a patient of the Mount Gambier Hospital (in rural South Australia) either as an inpatient or an outpatient, or a client of the local community care organization that delivered the program. Patients were not invited if currently physically or mentally distressed (eg by acute illness) where participation in the study would be burdensome. Participants were also required to use a simple mobile phone or Internet-based program. Mobile phones and dongles were provided for those participants who were experienced in the use of mobile phones and the Internet but who did not currently have access. Patients would be approached for recruitment after case note review, in medical or surgical wards of the hospital, in outpatient clinics, in the emergency department, and in community care settings. Potential participants were approached by nursing staff in the hospital and by community care workers in the community. The study was approved by the government of South Australia, Department of Health, Department of Health Human Research Ethics Committee.

### Training in the Program

Health care workers had existing credentials and experience in delivery of the Flinders Program but had no knowledge of goACT prior to the study. The health care workers were provided with initial training in goACT and follow-up support. Ongoing modifications were made to the health care worker interface and functions in response to in-use experiences.

The health care worker introduced each participant to the goAct software on an Internet-enabled computer at the care organization or on a mobile phone that had an Internet connection. Ongoing technology-related education was provided by the health care worker at face-to-face visits and/or over the phone to match the learning needs of the participant.

### Quantitative: Measures of Online Activity

The goACT program had the capacity to record a range of on line activity of subjects, including the number and date of subject logins.

### Quantitative: Outcome Measures

At baseline, a range of sociodemographic and diagnostic data were recorded. Subjects also completed the SF36 [18], the K10 [19], and the Partners in Health scale [20]. These scales were repeated at 6 months.

### Qualitative Assessment

Qualitative findings were drawn from documentary records. These were notes recorded by the health care worker conducting recruitment at Mount Gambier Hospital and a study report written by the health care worker responsible for delivery of the intervention program. This narrative report was based on the experiences and observations of the two health care workers delivering the program, along with findings from exit interviews performed by the workers as participants left the program. The content of the notes and report were analyzed using established thematic analysis methods to derive the two themes of feasibility of use and acceptability of use, and the subcategories such as “infrastructure/hardware problems” and “IT skills and confidence” [21,22].

We report quantitative and qualitative findings for recruitment (as an indicator of patient acceptance), use of the system by patients and clinicians (as indicators of engagement and feasibility of delivery), and outcome measures (as an indicator of potential effectiveness).

## Results

### Recruitment: Quantitative Findings

We recruited 8 participants to the program during the study: 5 from medical and surgical wards of the Mt Gambier Hospital and 3 from community services. The profiles of subjects recruited into the study are shown in Table 1 and their online interactions are shown in Table 2.

**Table 1:** Participant demographic data and diagnosis.

Case	Gender	Age (y)	Marital status	Source of referral	Diagnosis/conditions
1	M	54	Not known	A&E^a^/hospital	Diabetes, multiple sclerosis
2	F	63	Married	A&E/hospital	Diabetes, post-traumatic stress disorder, hypertension, tachyarrhythmia
3	M	78	Married	Mental health team	Depression, back pain, eyesight, low mobility
4	F	65	Not known	A&E/hospital	Diabetes type 2, fibromyalgia, hypercholesterolemia, hypertension, diabetic neuropathy, restless legs, low mobility
5	F	51	Not known	A&E/hospital	Chronic pain, social agoraphobia
6	M	62	Not known	General practitioner	Depression, Crohn disease, osteoarthritis, anemia, bipolar disorder
7	F	70	Not known	Psychosocial rehabilitation service	Bipolar disorder
8	F	49	Single	Employment access (disability employment) service	Fibromyalgia, depression, scoliosis

^a^A&E/hospital: Accident and emergency department in a hospital.

**Table 2:** Participants’ online interactions.

Interaction (No. of patients)		Mean (SD; SEM)
Number of logins (8)		26.4 (23.5; 8.3)
**Communications**		
	emails to patients (4)	1.8 (0.5; 0.3)
	SMS to patients (3)	1.7 (0.6; 0.3)
	Internal emails sent to patients (7)	3.9 (3.4; 1.3)
	Internal emails sent by patients (2)	4.5 (2.1; 1.5)
**Diary entries**		
	Exercise (3)	17.3 (19.6; 11.3)
	Mood (5)	11.2 (14.3; 6.4)
	Notes (3)	9.7 (13.3; 7.7)
**Activities tracked**		
	Not done (8)	16.5 (18.9; 6.7)
	Completed (8)	16.5 (15.2; 5.4)
	Completion rate (6) (%)	45.2 (26.3; 10.7)

**Figure 1:**
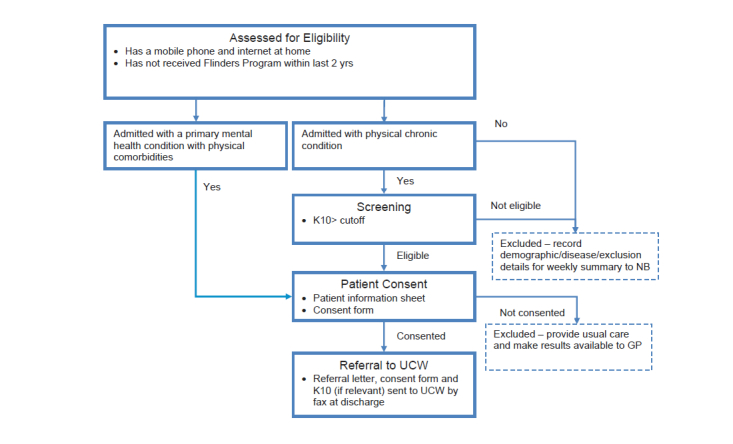
Participant recruitment process.

**Figure 2:**
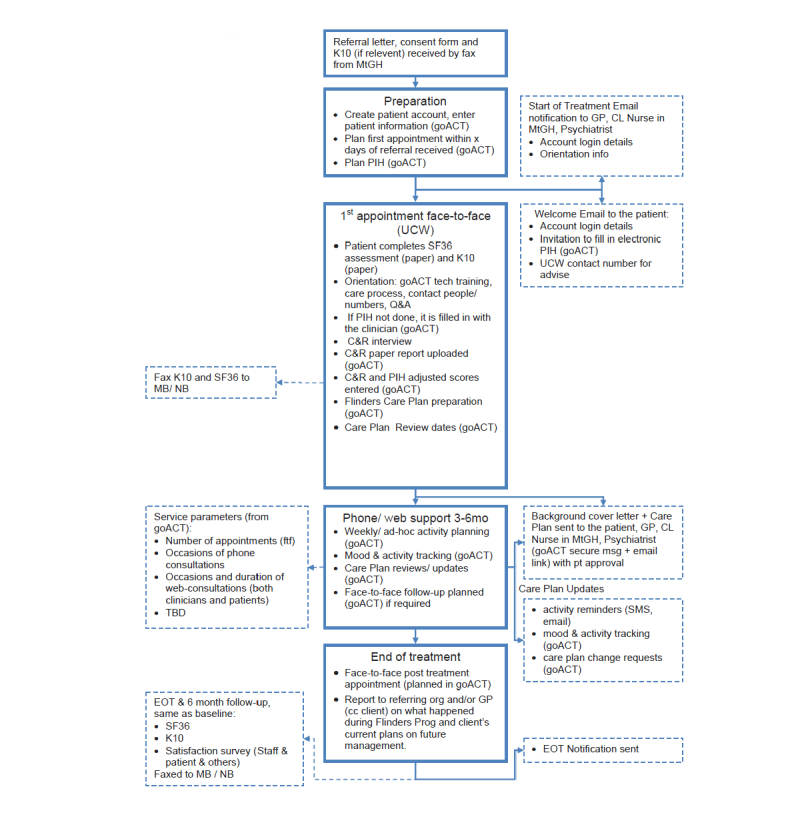
Intervention process.

During the recruitment period, 16 inpatients were identified from notes as meeting inclusion criteria, and 12 (75%) of these were available when study staff were available to conduct recruitment (eg, not transferred to another hospital or died). Of the 12, 5 (42%) consented and participated, 3 (25%) declined, and 3 (25%) initially consented but withdrew before participating. A further 3 patients were approached from community services, all of them consented and participated. The recruitment and intervention processes are outlined in Figures 1 and 2.

### Recruitment: Qualitative Findings

The health care workers' narrative report of the project commented on unexpected difficulties with recruitment. Severity of illness was the main reason that approaches were not made. The report noted that there were unexpectedly high numbers of younger, more acutely ill patients admitted to the hospital during the period of the study, thereby reducing the number of patients who fit the inclusion criteria due to the severity of illness. The study report also noted that recruitment from the emergency department was not feasible due to high levels of acuity, lack of privacy to discuss the study project, and pressure for patients to be quickly triaged. Those who were approached but declined gave a range of reasons, including stigma associated with participating in a project associated with psychological health.

### System Use: Quantitative Findings

All participants owned a mobile phone or had Internet access except for 2 participants, who were provided with a mobile phone and dongle to enable participation.

Overall, the goACT online management program was accessed 383 times during the study period. The 2 health care workers logged in 172 times (169 by the main health worker for the study). There were 211 logins by 6 of the 8 participants and no logins by 2 participants (see breakdown in Figure 4 below). The median number of logins for each week after recruitment and total number of participants logging in per week are also shown in Figure 5. The number of logins varies between 4 and 8 per week during the first 8 weeks and decreases rapidly after this period. Table 2 contains descriptive statistics for different types of interactions with patients.

The average number of logins by day of the week is shown in Figure 4.

**Figure 3:**
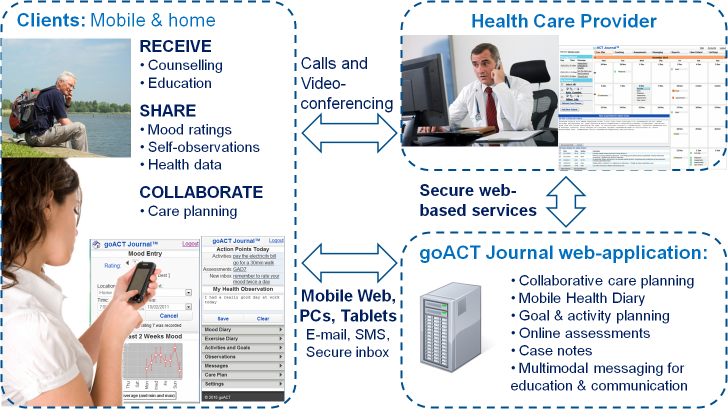
goACT platform.

**Figure 4:**
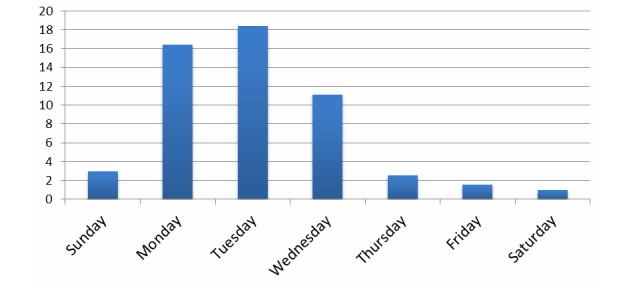
Average number of logins using goACT.

**Figure 5:**
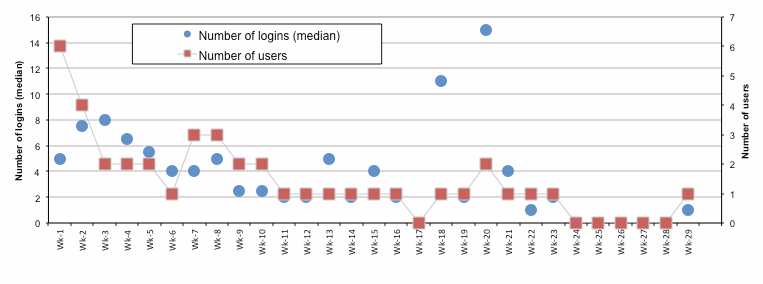
Daily login pattern.

### System Use by Health Care Workers: Qualitative Findings

The narrative study report provided the views and experiences of the health care workers in relation to the feasibility, acceptability, and development issues of delivering the Flinders Program via the goACT platform.

The health care worker was able to use goACT online management program to manage delivery, record-keeping, and communication with patients and other health professionals for the Flinders Program. However, Flinders Program aspects requiring in-depth conversations between health care worker and patient were entered into the software after the consultation. This was deemed necessary by the health care worker to achieve effective and conversational face-to-face interaction. This is important because the Flinders Program is based on patient-centered communication and shared decision-making.

Staff reported that the software was beneficial in that it supported electronic scoring of problems, goals, and activities. This was more useful than the traditional scoring on paper copies because it removed any need for transcribing. For example, goACT increased the ease of sharing information with other staff, including general practitioners who were not in the same area.

Despite the difficulties that the pilot group of participants experienced with the e-version of the program (as explained in the next section), the health care workers thought that the ability to deliver the Flinders Program via goACT added extra layers to the communication between staff and patient. They thought that there could be more attractive or convenient ways for some patients to interact with their health care workers, or for the patients to be more involved in their self-management. The goACT program clearly offers new communication options via email, as well as tools such as an exercise diary and the capacity to provide links to high-quality disease information and online therapy websites.

### System Use by Patients: Qualitative Findings

In the narrative report, health care workers observed that most participants encountered difficulties with technology use and none used the software extensively. Information technology issues fell into three different categories. First, infrastructure/hardware problems, including drop-out of rural Internet connections (which are still not highly reliable in all areas of rural Australia) and 1 participant's mobile phone being stolen. It was difficult for staff to complete the C&R and P&G with 1 participant as they were repeatedly logged off the Internet. This problem was later resolved by obtaining a more efficient Internet dongle. Physical barriers to use included small-sized phone screens and poor eyesight. The second problem was related to general information technology (IT) skills and confidence. Client difficulties with using the FP+goACT software reflected their general lack of basic knowledge and confidence for use of IT. Participants may have had general lack of familiarity with mobile phones and the Internet or limited experience (eg, familiarity with voice calls on a mobile phone but not SMS or Internet access). Third, the degree of illness severity affected performance. For 2 participants, their health conditions affected their ability to use the software. For example, due to multiple sclerosis, 1 participant reported having difficulty comprehending and remembering information. This reduced his ability to use the goACT software because he was unable to recall how to use the software after training from both the health care worker and his wife.

### Outcome Measures: Quantitative Findings

Differences between scores at admission and exit were analyzed using paired t-test, in SPSS version 19. There was little change in functional outcome during the period of the study, as indicated in Table 3.

**Table 3:** Functional outcome changes in health.

	Admission to Program	Exit from Program	Change		
Scale	Mean, SEM, SD	Mean, SEM, SD	Mean, SEM, SD	Student’s *t* test	*P* value
PIH^a^	71.4, 3.7, 8.2	66.8, 3.5, 7.8	4.6, 5.0, 11.1	0.92	0.4
K10^b^	32.2, 2.6, 5.8	30.8, 2.4, 5.3	1.4, 1.1, 2.5	1.25	0.3
SF36 (Bodily pain score)^c^	37.8, 12.5, 28.0	43.6, 5.1, 11.5	−5.8, 11.6, 25.9	−0.50	0.6
SF36 (Emotional score)^c^	13.3, 8.2, 18.3	26.7, 12.5, 27.9	−13.3, 17.0, 38.0	−0.78	0.5
SF36 (Physical functioning score)^c^	29.0, 12.4, 27.7	22.0, 9.3, 20.8	7.0, 3.7, 8.4	1.87	0.1
SF36 (Social functioning score)^c^	40.0, 10.8, 24.0	62.5, 11.9, 26.5	−22.5, 14.5, 32.4	−1.55	0.2
SF36 (Mental health score)^c^	50.4, 6.0, 13.4	46.4, 5.9, 13.1	4.0, 5.5, 12.3	0.73	0.5
SF36 (Physical health summary)^c^ using Australian norms	31.8, 7.8, 17.5	29.2, 3.0, 6.6	2.6, 5.3, 11.8	0.49	0.7

^a^Range, 0-96.

^b^Range, 0-100.

^c^Range, 10-50.

## Discussion

This pilot study has demonstrated that an existing chronic disease management program can be successfully transferred to an existing eHealth platform for combined face-to-face and eHealth delivery. It also provides pointers for further development and targeting of eHealth-facilitated chronic disease management.

After training, the 2 health care workers used the goACT platform to successfully manage and deliver the Flinders Program, although a significant amount of additional time and effort was required for the health care worker to become familiar and skilled with goACT. They reported advantages to the eHealth version over the traditional paper-based program, such as greater ease of sharing patient information with other health care professionals. The health care workers proposed however that the eHealth version piloted was useful as an additional layer in service delivery, but not as a complete replacement.

The rapid decrease in number of weekly logins after the first 8 weeks (Figure 3) might be explained by the decrease in intensity of coaching that occurs during the later parts of the Flinders care plan. It is encouraging to note that the automated weekly care summary email sent each Sunday night was associated with a higher number of logins in the earlier part of the week. The email summarized care plan activities, appointments, and data provision scheduled for the upcoming week and reminded participants that they could go into the system to see more detail and check off completed activities. The emails could be switched off by participants but none chose to do so. Future research in eHealth interventions should focus on strategies to maintain engagement beyond the early period, such as optimizing automated and personalized online support.

The intervention achieved only limited participant use. One reason may be that most participants had complex and severe illnesses and their daily lives were concerned with managing their health conditions. As well as health status, other factors limiting successful eHealth use among study participants may be the age profile (49-78 years old, with the average in their 50s or 60s), and the rural location of residents. These factors are consistently associated with lower levels of IT use in Australia [23,24]. These factors would limit their ability to be interested in, or successfully deal with, the addition of an unfamiliar eHealth program. We suggest that future studies of this kind might include initial screening using an e-literacy tool [25]. This would allow assessment of participants’ needs for support to use the hardware and software, thereby increasing the likelihood of success with the eHealth program, and identification of those patients requiring continuation with the offline version of the program. It would also be useful to identify the extent to which basic IT use and/or IT use for health self-management are barriers to engagement. In a study N=2,928, adults living with chronic disease (n=538) were less likely to go online (51%) than those without such disease (n=2,367) (74%), but once online, they were avid consumers of health information [26].

Study limitations included the small sample size. While outcome measurement was demonstrated to be feasible, the inclusion of 8 participants was too small to demonstrate any changes in health-related measures.

In summary, the pilot study demonstrated the feasibility of implementing and delivering a chronic disease management program using a Web-based patient-clinician application in a rural setting. If initial barriers to IT use can be addressed, then people with chronic conditions can be successful users of eHealth systems such as FP+goACT.

## Abbreviations

C&R: cue and response interview
IT: information technology
PIH: partners in health scale
P&G: problem and goals assessment
SMS: short message service
